# A Comparative Analysis of Age, BMI, Age/BMI Ratio, Nottingham Hip Fracture Score, and American Society of Anesthesiologists (ASA) Grade as Predictors of 30-Day Mortality After Neck of Femur Fractures: A Retrospective Cohort Study

**DOI:** 10.7759/cureus.73021

**Published:** 2024-11-04

**Authors:** Matthew Goodman, Anand Pillai

**Affiliations:** 1 Trauma and Orthopaedics, University of Manchester, Manchester, GBR; 2 Trauma and Orthopaedics, Wythenshawe Hospital, Manchester, GBR

**Keywords:** hip fracture, mortality, orthopaedics, risk, trauma

## Abstract

Background and objective

Hip fracture is a condition associated with high mortality rates, necessitating the use of risk assessment tools to optimise patient care. This study aimed to introduce and describe a novel score using Age/BMI as an improved predictor of 30-day mortality

Methods

A retrospective cohort study was conducted at a high-volume neck of the femur centre. Data from 574 patients treated over one year were collected and analysed. Multivariate logistic regression analysis was used to determine variables that significantly increased the risk of 30-day mortality.

Results

A total of 574 patients were identified: 388 females and 186 males. The overall mortality of the patient cohort at the time of data collection was 21.78% (n=125). The 30-day mortality was found to be 5.75% (n=33) while the one-year mortality rate was 21.08% (n=121). The key risk factors for mortality in neck of femur fractures, highlighted in the literature review, were compared against the binomial outcome variable of 30-day mortality. Categorical data analysis was first completed to highlight key trends. A regression analysis then demonstrated the significance of each factor. Age (p=0.75207), BMI (p=0.97674), and Age/BMI (p=0.92205) showed no statistical significance. The Nottingham Hip Fracture Score (NHFS) was marginally significant (p=0.05749). The American Society of Anesthesiologists (ASA) grade was shown to be statistically significant, emerging as the strongest predictor of 30-day mortality (p=0.00953).

Conclusions

Our findings show that current guidelines utilising ASA and NHFS are excellent predictors of 30-day mortality in hip fracture patients. The proposed Age/BMI score did not demonstrate efficacy in this cohort. Further research is warranted to explore alternative predictors and enhance risk assessment in this population.

## Introduction

The neck of femur fracture, a form of hip fracture, refers to a break in the proximal femur between the superior margin of the femoral head and 5 cm below the lesser trochanter. These fractures can be categorised into two main groups: intracapsular (those occurring above the insertion point of the joint capsule) and extracapsular (where the fracture occurs below the insertion point). Extracapsular fractures can be classified further as trochanteric or subtrochanteric, based on the location of the fracture relative to the greater trochanter. While the management of hip fractures is highly variable, achieving full weight bearing in the postoperative period is invariably the major objective of the treatment. Factors that influence the treatment decisions include the location, fracture displacement, prior mobilisation, and fitness for surgery [1].

Hip fractures are of clinical relevance due to their poor outcomes, particularly mortality, with an estimated 10% 30-day mortality rate and one-third within one year [1]. According to the National Institute of Health and Care Excellence (NICE), 65,000 hip fractures occur each year on average, with an expected rise due to an ever-increasingly ageing population. Hip fracture is associated with annual hospital costs of £1 billion in the UK, without considering the further financial impact of social care [1]. The National Hip Fracture Database (NHFD) 2023 Report has highlighted the rising number of reported hip fractures. The document states that there were a total of 72,160 hip fractures in 2022, compared with less than 66,000 in both 2020 and 2021 [2]. Several studies have observed that patient demographics, management choice, and socioeconomic factors all contribute to mortality in this cohort [3]. Sheehan et al. reported a total of 39 different factors that may contribute to the high hip fracture mortality rate seen in neck of femur fractures [4]. These figures for mortality are further complicated by the fact that many deaths from hip fractures occur not only due to the break itself but also as a result of various other factors and comorbidities in the perioperative period [1].

Preoperative recognition of high-risk patients who tend to have poor outcomes could help reduce the overall mortality rates in neck of femur fractures. This enables priority access to surgery lists and improved care both in the perioperative and postoperative periods, potentially leading to improved quality of life as well as lower mortality. Such risk stratification tools are routinely used in medicine and may involve single-factor or multi-faceted scoring systems. Our initial literature search for predictors of mortality in the neck of femur fractures revealed several retrospective studies exploring these systems, and the examples of cumulative systems include POSSUM, Nottingham Hip Fracture Score (NHFS), the Charlson Comorbidity Index, and Sernbo score [5-9]. Other single-factor predictors have also been suggested, such as the American Society of Anesthesiologists (ASA) score, Albumin, CRP, Haemoglobin, and comorbidities [10-14]. This is a widely debated area with a large number of suggested theories. One reason for the difficulty in establishing a single mortality predictor is the broad range of confounding patient variables, as discussed previously.

Current guidelines at the study hospital advocate for the use of NHFS and the ASA score [15]. NHFS is a tool comprising seven patient factors, which aims to predict 30-day mortality [16]. It has also been employed as a predictor of one-year mortality in a separate study [7]. The score ranges from 0 to 14 with a median of 4 [16]. NHFS considers age (66-85 and ≥86 years), sex (male), cognitive function [Abbreviated Mental Test Score (AMTS) ≤6], haemoglobin on admission (≤100 g/l), prior residence, number of comorbidities (≥2), and active malignancy in the last 20 years [8]. The ASA score assesses pre-anaesthesia comorbidities to help predict a patient’s perioperative risks [17]. It ranges from 1 (a normal healthy patient) to 6 (a declared brain-dead patient whose organs are being removed for donor purposes) with the score progressing based on comorbidities and functional limitations [17].

Age and BMI have been consistently mentioned as risk factors for mortality in the literature [3,10,18,19]. A study by Al Balwi et al. combined Age and BMI as a potential predictor of mortality in coronavirus disease 2019 (COVID-19) patients [20]. A higher BMI in the elderly population was shown to have beneficial effects in reducing mortality [20]. Another study drew on the findings of the above-mentioned research to analyse the postoperative mortality of cholecystectomy patients [21] and demonstrated that a higher Age/BMI score was associated with an increased risk of mortality, in a cohort of 435,052 patients [21]. Several theories have been proposed to explain this finding; one of them states that patients with a low BMI are more likely to be frail, with a reduced ability to cope with the stress of surgery and recovery [19,21]. Similarly, the same effect can be seen in elderly patients, which tends to be linked with poorer surgical outcomes [22]. In light of these findings, this study aimed to devise a predictor of 30-day mortality in neck of femur fractures [20]. This will then be compared against the current standard involving the NHFS and the ASA grade.

## Materials and methods

A literature review was conducted prior to the study on the databases PubMed and The University of Manchester Library. The following search terms were used in various combinations: "neck of femur fracture, hip fracture, mortality, predictor, age, BMI, and risk factor". Articles were then selected based on their relevance to the topic, and information was extracted and assimilated. The study was conducted at Wythenshawe Hospital, a high-volume centre for neck of femur fractures. This was a retrospective cohort study performed to analyse the hospital data of patients presenting in 2023. The list of potential participants was collected from NHFD, a nationwide audit within the NHS concerning hip fracture management and outcomes. This initial cohort included 589 patients. HIVE electronic patient records system was used to access further patient demographics, mortality outcomes and NHFS variables. This initial data collection documented patient data regarding age, sex, BMI, ASA grade, admission haemoglobin, comorbidities, malignancy, AMTS, and primary residence.

All data were entered into a Microsoft Office Excel Spreadsheet. A formula was then generated to calculate NHFS in retrospect (Table 9 in Appendices). ASA grade was recorded by the anaesthetist and included within the NHFD (Table 10 in Appendices). BMI information was collated from surgical records or admission data. The age of the patient at the point of presentation was recorded. The main outcome measure of this study was death following a neck of femur fracture. Mortality was grouped into three categories: within 30 days, within one year and beyond one year. The 30-day mortality was used for analysis as this constituted the most accurate figure for the 2023 dataset at the time of writing. The exclusion variables included overseas patients (n=6), no recorded ASA score (n=7), and no recorded BMI (n=2); a total of 15 patients were excluded from the analysis.

Patient subgroups were created, as determined by specific variables to analyse the effect of each upon 30-day mortality. This provided an initial understanding of the distribution of 30-day mortality against the individual variables of age, BMI, Age/BMI, NHFS, and ASA grade. R software was used to produce a model of logistic regression. The binomial outcome variable of 30-day mortality (yes/no) was compared against the following factors: age (continuous), BMI (continuous), Age/BMI (continuous), NHFS (categorical), and ASA grade (categorical). The coefficient estimate, standard error, Z value, and p-values were calculated.

## Results

A total of 574 patients were identified: 388 females and 186 males. Table 1 summarises the demographic data of our study cohort. The management of patients is summarised in Table 2.

**Table 1:** Demographics data of the cohort (N=574) ASA: American Society of Anaesthesiologists; BMI: body mass index; Hb: haemoglobin; NHFS: Nottingham Hip Fracture Score; SD: standard deviation

Demographics	Values
Age, years	
Mean ± SD (range)	80.67 ± 9.05 (60–102)
Sex, n (%)	
Female	388 (67.60%)
Male	186 (32.40%)
ASA score	
Mean ± SD (range)	3.08 ± 0.63 (1–4)
NHFS	
Mean ± SD (range)	4.84 ± 1.62 (0–9)
BMI, kg/m^2^	
Mean ± SD (range)	23.42 ± 5.55 (10.82–47.45)
Comorbidities, n (%)	
Chronic kidney disease (CKD)	96 (16.72%)
Diabetes	89 (15.51%)
Ischaemic heart disease (IHD)	46 (8.01%)
Dementia	55 (9.58%)
Arthritis	74 (12.89%)
Hb on admission, mean ± SD	121.63 ± 17.37
Living in an institution, n (%)	72 (12.54%)

**Table 2:** Management options HA: hemiarthroplasty; hemi: hemiarthroplasty; IM: ntermedullary; THR: total hip replacement

Management, n (%)	
Arthroplasty - bipolar hemi (cemented)	98 (17.07%)
Arthroplasty - bipolar hemi (uncemented - HA-coated)	5 (0.87%)
Arthroplasty - THR (cemented)	1 (0.17%)
Arthroplasty - THR hybrid	25 (4.36%)
Arthroplasty - Unipolar hemi (cemented/modular)	5 (0.87%)
Arthroplasty - unipolar hemi (cemented/monoblock)	140 (24.39%)
Arthroplasty - unipolar hemi (uncemented - HA-coated/monoblock)	1 (0.17%)
Arthroplasty - unipolar hemi (uncemented - uncoated/monoblock)	6 (1.05%)
Internal fixation - cannulated screws	13 (2.26%)
Internal fixation - IM nail (long)	70 (12.20%)
Internal fixation - IM nail (short)	41 (7.14%)
Internal fixation - IM nail with plate and screws combined	1 (0.17%)
Internal fixation - sliding hip screw	140 (24.39%)
Other	20 (3.48%)
Conservative	8 (1.39%)

The overall mortality of the patient cohort at the time of data collection was 21.78% (n=125). We observed a 30-day mortality of 5.75% (n=33) and a one-year mortality of 21.08% (n=121). An initial categorical analysis of the data was completed. Age was broken into brackets of 10 years, with ages over 89 years placed in a single cohort. The brackets were as follows: 60-69, 70-79, 80-89, and >89 years (Table 3). BMI was grouped under the NHS-suggested brackets of Underweight, Normal, Overweight, Obesity, and Severe Obesity (Table 4) [23]. The Age/BMI factor was grouped into trivial brackets between set integers for initial analysis. This consisted of seven distinct brackets with results varying from 1.45 to 8.50, to two significant figures (Table 5). NHFS data constitute categorical data ranging from 0 to 9 (Table 6). Finally, the ASA grading was given as integers, with results ranging between 1 and 4 (Table 7). The percentage of mortality rate for each category was calculated to two significant figures, allowing a broad assessment of trends within the chosen risk factors of mortality.

**Table 3:** Age of patient cohort against 30-day mortality

Age, years	30-day mortality			
	No	Yes	Grand total	Percentage of death rate
60-69 years	78	1	79	1.28
70-79 years	164	8	172	4.87
80-89 years	209	13	222	6.22
>89 years	90	11	101	12.22
Grand total	541	33	574	-

**Table 4:** BMI of patient cohort against 30-day mortality BMI: body mass index

BMI categories	30-day mortality			
	No	Yes	Grand total	Percentage of death rate
Underweight	91	5	96	5.49
Normal	271	19	290	7.01
Overweight	119	6	125	5.04
Obesity	50	3	53	6.00
Severe obesity	10	0	10	0
Grand total	541	33	574	-

**Table 5:** Age/BMI of patient cohort against 30-day mortality BMI: body mass index

Age/BMI	30-day mortality			
	No	Yes	Grand total	Percentage of death rate
1≤X<2	17	0	17	0
2≤X<3	130	8	138	6.15
3≤X<4	227	10	237	4.41
4≤X<5	118	11	129	9.32
5≤X<6	41	4	45	9.76
6≤X<7	7	0	7	0
7≤X<8	0	0	0	-
8≤X<9	1	0	1	0
Grand total	541	33	574	-

**Table 6:** NHFS of patient cohort against 30-day mortality NHFS: Nottingham Hip Fracture Score

NHFS	30-day mortality			
	No	Yes	Grand total	Percentage of death rate
0	12	0	12	0
1	13	0	13	0
2	10	0	10	0
3	62	0	62	0
4	108	1	109	0.93
5	168	15	183	8.93
6	104	11	115	10.58
7	44	3	47	6.82
8	13	2	15	15.38
9	7	1	8	14.49
Grand total	541	33	574	-

**Table 7:** ASA grade of patient cohort against 30-day mortality ASA: American Society of Anesthesiologists

ASA grade	30-day mortality			
	No	Yes	Grand total	Percentage of death rate
1	5	0	5	0
2	74	1	75	1.33
3	344	16	360	4.65
4	117	16	133	13.68
Grand total	541	33	574	-

The result of the logistic analysis of each variable is shown in Table 8.

**Table 8:** Results of logistic regression analysis* *Performed using R Software ASA: American Society of Anaesthesiologists; BMI: body mass index; NHFS: Nottingham Hip Fracture Score; RC: regression coefficient; SE: standard error

Variable	RC	SE	Z value	P-value
Intercept	-7.984	3.189	-2.503	0.012
Age at presentation	0.0119	0.0377	0.316	0.752
BMI	-0.0027	0.0926	-0.029	0.977
Age/BMI cofactor	-0.0577	0.5896	-0.098	0.922
NHFS	0.297	0.1566	1.896	0.058
ASA grade	0.8893	0.343	2.592	0.010

## Discussion

We performed this study to assess the risk of death after hip fracture at a single high-volume centre. Our initial review of the literature showed an estimated 30-day mortality of 10% (NICE), which is considerably higher than the 5.75% recorded in this study [1]. One year mortality was recorded as 21.08%, lower than that recorded by NICE (33%); however, it could not be fully assessed as it contained 2023 data [1]. Instead, this can only be quantified from the start of 2025.

The initial evaluation of subgroups revealed some trends in mortality rates. Within the designated age brackets, a positive correlation was seen between increasing age and increasing percentage of death rate in the group. This suggests that age is a risk factor for mortality; however, this finding cannot be considered to be of major significance due to the use of trivial brackets. BMI showed no obvious impact on mortality outcomes. The theorised Age/BMI factor was grouped into trivial brackets to assess for interdependence, and hence the analysis is less reliable. No linear relationship was seen between the subgroups and mortality with a 0% death rate at both ends of the parameter. However, a proportionately higher death rate was noted in the fourth and fifth groups: 9.32% and 9.76% respectively.

NHFS alluded to an association between a high score and mortality rate, which may indicate that it is an effective predictor of mortality. Survival curves of NHFS have shown a divergence at scores greater than 4, distinguishing between high- and low-risk patients [8]. Integrating our data into this model was highly supportive, with only one death in patients in the low-risk group (n<4). While variation was seen between the high-risk groups, the highest percentage rates were seen in the top two brackets. Lastly, a strongly positive association was observed between ASA grade and mortality in this cohort. The initial evaluation may indicate that this parameter is a useful predictor of 30-day mortality in hip fracture patients.

Logistic regression showed that the coefficient of age was not statistically significant (p=0.75207). This suggests that the variable may not have a significant effect on outcome. BMI was shown to have a p-value of 0.97674, which does not indicate a significant effect on the 30-day mortality. Similarly, Age/BMI was not shown to be a significant predictor of outcome in this patient cohort (p=0.92205). NHFS was found to be marginally significant with p=0.05794, which is greater than the confidence interval of less than 0.05 for highly predictive factors, but within the interval of p<0.1. NHFS was indicated to have some association with the outcome variable; however, further investigation is warranted due to the borderline result observed. ASA grade was shown to be the only statistically significant predictor of the binomial outcome variable (p=0.00953).

Our study findings contrast with those by Al Balwi et al. and Fakhoury et al., stating that Age/BMI is not a strong predictor of 30-day mortality [20,21]. When applied to COVID-19 and cholecystectomy patients, the use of Age/BMI was found to be useful; however, these results suggest that it may not be possible to replicate its application in other clinical conditions. A possible reason for this is that neither age nor BMI as separate indicators were shown to be strongly linked with mortality in this population group. This is contrary to the findings of the initial literature review, which suggested that these factors are correlated, theorising the production of a combined score. Further study is required to assess whether Age/BMI has the potential to be used as a mortality predictor in other hip fracture cohorts.

Al Balwi et al. highlighted that increasing BMI serves as a protective factor against frailty among the elderly population. Increased frailty has been consistently linked to higher mortality rates, making this protective effect particularly significant. Their research, encompassing a broader age demographic, facilitated comparison between younger and elderly populations. However, the limited age range in our study inhibits a direct comparison of mortality rates, preventing a conclusive determination of whether a low BMI truly poses a significant risk among elderly patients. Data correlate with the current guidelines that NHFS and ASA grades have a stronger association with 30-day mortality, endorsing the literature that reinforced using these classifications in hip fractures. NHFS was developed in a study with a large sample and has been supported by further investigation and implementation in several hospitals. To further analyse NHFS, it would be beneficial for it to be included in NHFD. This would enable a definitive assessment of the mortality predictor throughout the UK, as part of the national audit. Even though it is in the current guidelines, we could not gather the results and it required the collation of each of the individual factors.

ASA grade was the only measure that demonstrated a significant correlation (p<0.05). This suggests that it is the superior of the two measures. A possible reason for this may be the element of holistic assessment, considering multiple factors including comorbidities, obesity (BMI), functional limitations, and substance use. These were all shown to be risk factors for mortality in hip fracture, suggesting that it correlates well with predicting outcomes in this population. One disadvantage of the ASA grade is associated with its use in conservative management. As a perioperative assessment tool, it may not be properly employable in patients who do not undergo surgery.

The outcomes of this study endorse the continued use of NHFS and ASA grades in clinical practice. Continued monitoring and quality improvement within the field will enable ongoing refinement and validation of these risk assessment tools. However, it would not be advisable to suggest the use of Age/BMI in healthcare decision-making.

Limitations

This study has two main limitations, linked to its methodology. This was a single-centre study over the course of one year. Although practice and patient cohorts are likely representative of the UK as a whole, there may be aspects that differ from other hospitals. The application of the exclusion led to the removal of 15 patients from data analysis. However, since it makes up only a small proportion of the original data set (2.55%), we believe it will not have a significant effect on the outcomes of the study. It should be noted that three patients were reported deceased, not including the overseas patients.

## Conclusions

This study sheds light on the predictors of mortality in neck of femur fractures, underlining the importance of accurate risk assessment and targeted interventions in clinical practice. Our findings show that Age/BMI is not a useful measure of 30-day mortality. Instead, the continued use of current guidelines, in particular ASA grade, is recommended. This was a small cohort study at a single hospital, and we recommend that the measure be tested on a larger cohort of individuals. The incorporation of NHFS and BMI into NHFD could facilitate this. Documenting mortality data, including specific one-year mortality rates, may prove to be advantageous in long-term risk assessment. Continued research is crucial to enhance risk prediction, aiming to mitigate the high mortality rate associated with hip fractures.

## Appendices

**Table 9:** Calculation of Nottingham Hip Fracture Score* *[16] AMTS: Abbreviated Mental Test Score; Hb: haemoglobin

Parameter	Criteria	Score
Age, years	66–85	3
	>85	4
Sex	Male	1
AMTS	<7/10	1
Hb on admission	<100 g/l	1
Residence	Living in an institution	1
Comorbidities	Greater than 2	1
Active malignancy	Within the last 20 years	1
Total		Max 10

**Table 10:** Current definitions and ASA classifications* *[17] ARD: acute renal disease; ASA: American Society of Anaesthesiologists; BMI: body mass index; CAD: coronary artery disease; COPD: chronic obstructive pulmonary disease; CVA: cerebrovascular accident; DIC: disseminated intravascular coagulation; DM: diabetes mellitus; ESRD: end-stage renal disease; HTN: hypertension; MI: myocardial infarction; TIA: transient ischaemic attack

ASA physical status classification	Definition	Adult examples, including, but not limited to:
ASA I	A normal healthy patient	Healthy, non-smoking, no or minimal alcohol use
ASA II	A patient with mild systemic disease	Mild diseases only without substantive functional limitations. Current smoker, social alcohol drinker, pregnancy, obesity
ASA III	A patient with severe systemic disease	Substantive functional limitations; one or more moderate to severe diseases. Poorly controlled DM or HTN, COPD, morbid obesity (BMI ≥40), active hepatitis, alcohol dependence or abuse, implanted pacemaker, moderate reduction of ejection fraction, ESRD (undergoing regularly scheduled dialysis), history (>3 months) of MI, CVA, TIA, or CAD/stents
ASA IV	A patient with severe systemic disease that is a constant threat to life	Recent (<3 months) MI, CVA, TIA or CAD/stents, ongoing cardiac ischemia or severe valve dysfunction, severe reduction of ejection fraction, shock, sepsis, DIC, ARD or ESRD (not undergoing regularly scheduled dialysis)
ASA V	A moribund patient who is not expected to survive without surgery	Ruptured abdominal/thoracic aneurysm, massive trauma, intracranial bleed with mass effect, ischemic bowel in the face of significant cardiac pathology or multiple organ/system dysfunction
ASA VI	A declared brain-dead patient whose organs are being removed for donor purposes	-
